# Antibiotic-functionalized gold nanoparticles for the detection of active β-lactamases†

**DOI:** 10.1039/d1na00635e

**Published:** 2021-12-13

**Authors:** Lisa M. Miller, Matthew D. Simmons, Callum D. Silver, Thomas F. Krauss, Gavin H. Thomas, Steven D. Johnson, Anne-Kathrin Duhme-Klair

**Affiliations:** Department of Chemistry, University of York Heslington York YO10 5DD UK lisa.miller@york.ac.uk; Department of Electronic Engineering, University of York Heslington York YO10 5DD UK; Department of Physics, University of York Heslington York YO10 5DD UK; Department of Biology, University of York Heslington York YO10 5DD UK

## Abstract

Antimicrobial resistance (AMR) continues to threaten the effective treatment and prevention of bacterial infections. The spread of resistant infections is accelerated by the lack of fast and cost-effective tests for the detection of AMR at the point-of-care. We aimed to address this challenge by developing a diagnostic tool to detect one of the major forms of AMR, the β-lactamase enzymes. Antibiotic-functionalized gold nanoparticles (AuNPs) have been successfully developed for the detection of β-lactamases in challenging biological media, namely undiluted urine. Furthermore, these tools are compatible with samples containing a urine sample preservative (boric acid) or hematuria (blood). The functionalized AuNPs interact with the active β-lactamases, resulting in the hydrolysis of the surface-bound antibiotics, which then inhibits binding of the AuNPs to a capture protein (a penicillin-binding protein) to indicate the presence of active β-lactamases. We successfully integrated the antibiotic-functionalized AuNPs into a new lateral flow assay (LFA), which can be used to detect active β-lactamases down to the detection limit of 11 nM. While we demonstrate the use of antibiotic-functionalized AuNPs in an LFA format to provide a novel method of detecting active β-lactamases, these functionalized AuNPs are amenable to a range of alternative diagnostic technologies and could lead to vital point-of-care diagnostics for the early detection of multi-drug resistant infections.

## Abstract 2

Antibiotic-functionalized gold nanoparticles able to selectively detect enzymatically-mediated antimicrobial resistance in undiluted urine.

## Graphical abstract

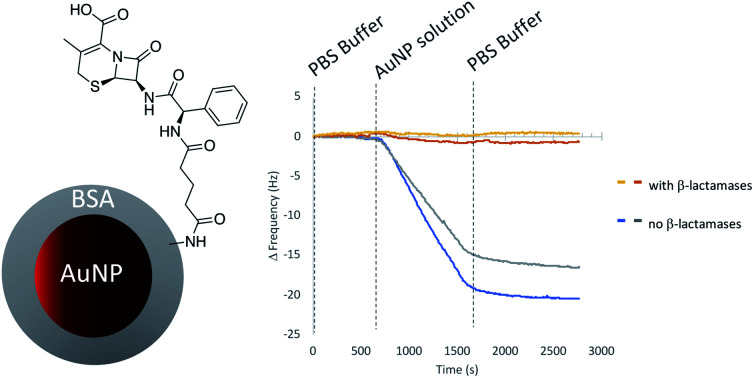

## Introduction

Antimicrobial resistance (AMR) is a well-recognized problem in the healthcare system.^1^ Attempts at combatting the continued spread of resistance and the emergence of new forms of resistance are fundamentally limited by our ability to detect the susceptibility of bacteria to antibiotics rapidly and at the point-of-care.^2^ A rapid test for the detection of resistance to a commonly prescribed class of antibiotics would provide clinicians with valuable information needed to inform treatment decisions.^3^

For example, bacteria are able to produce β-lactamases, enzymes that confer resistance to the β-lactam antibiotics (such as penicillin), which are one of the most commonly-prescribed classes of antibiotics.^4,5^ The extended-spectrum β-lactamases (ESBLs) pose a severe threat to human health, as these enzymes confer resistance to the more advanced antibiotics in this class, such as the third generation cephalosporins, thus limiting the effective treatments available for such infections.^6^ Furthermore, ESBL-producing organisms present with co-resistance to other classes of antibiotics, thus increasing the challenge of treating these infections.^7^

Currently, there is an unmet need for fast and cost-effective tests that provide point-of-care detection of β-lactamase activity and that can be used directly from a patient's sample. The gold standard test involves culturing bacteria present in a patient's sample and then assessing resistance profiles using disk susceptibility tests, a process which can take up to 72 hours.^3^ Several rapid tests for these enzymes are available commercially but the few that have been developed to work on unprocessed clinical samples (namely ESBL NDP Test, β LACTA™, and Rapid ESBL Screen kit 98022)^8–10^ require trained laboratory technicians and specialist equipment. The exception is the NG-Test CTX-M (Bio Trading)^11^ which is a simple-to-use lateral flow assay (LFA) that uses antibodies to recognize a specific family of β-lactamases present in bacterial isolates, but this test does not confirm the activity of the enzymes.

Previously, we reported the development of a surface-bound, clinically-relevant β-lactam drug that allowed for the detection of β-lactamases in undiluted urine.^12^ This work demonstrated that β-lactamases are able to recognize and react with the surface-bound drug molecules, thereby hydrolyzing the β-lactam. Hence, it was established that the surface-bound drug can be used to probe for/detect β-lactamase activity. Here, we develop these surface-bound antibiotics for application in biosensors, focusing on antibiotic-functionalized gold nanoparticles in an LFA format, that allows for the direct detection of β-lactamase activity. The LFA has been optimized for use in undiluted urine in order to improve management of urinary tract infections (UTIs), which has an estimated global prevalence of 150 million cases a year.^13^ UTIs are the second most common reason for the prescription of antibiotics^14^ and yet prescribing often takes place without prior testing to confirm the treatment efficacy. Therefore, a point-of-care UTI test that could identify drug-resistant infections would allow the right antibiotic to be chosen and prevent the misuse and overuse of antibiotics thus improving patient outcomes and helping to prevent the acceleration of antibiotic resistance.^15,16^

**Fig. 1:**
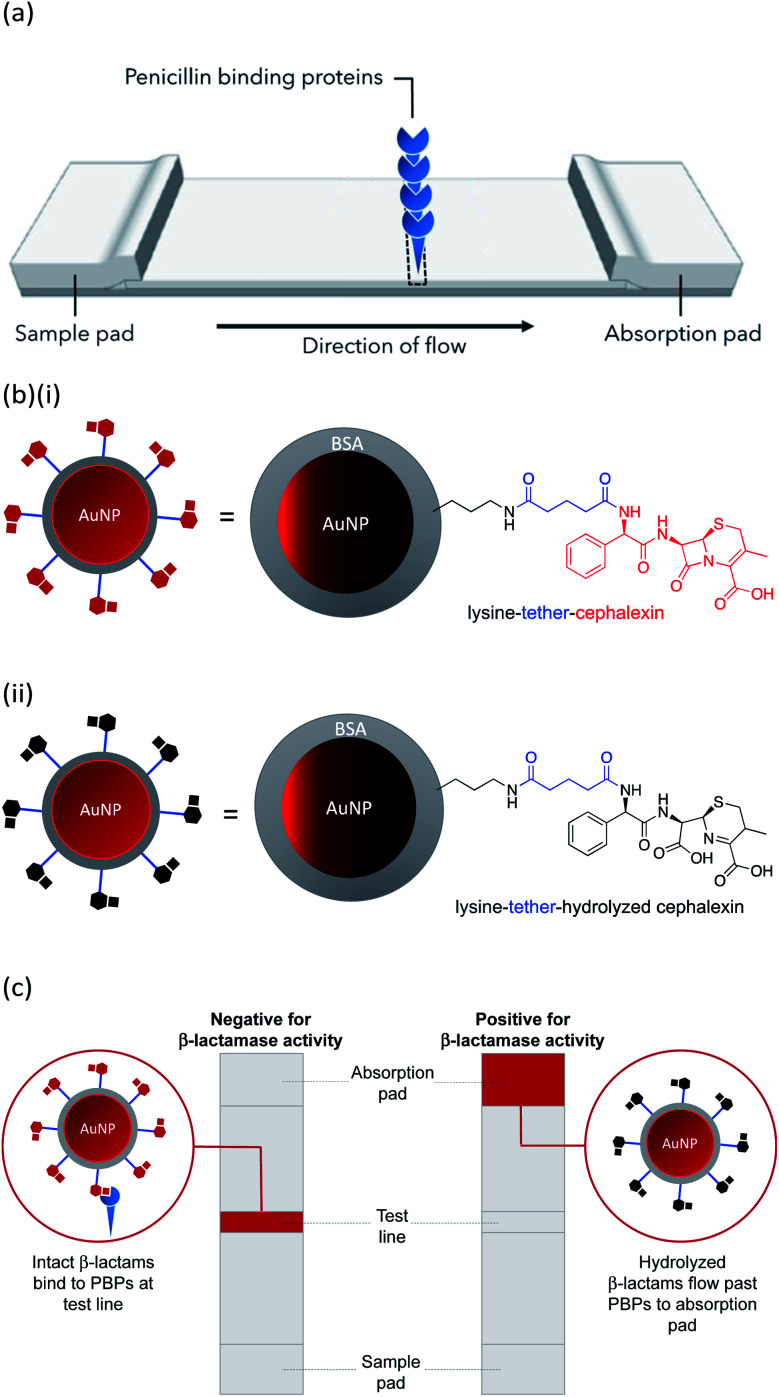
LFA using β-lactam antibiotic-functionalized AuNPs for the detection of β-lactamases: (a) design of LFA test strip; (b) β-lactam antibiotic-functionalized AuNPs: (i) intact β-lactam and (ii) hydrolyzed β-lactam; (c) test readouts of LFA. The final LFA also included a control line used to confirm a valid run of the assay.

In this work we describe the preparation of LFAs according to the design detailed in Fig. 1(a). Gold nanoparticles (AuNPs) labelled with a β-lactam antibiotic (Fig. 1(b)), act as probes by binding selectively to penicillin-binding proteins (PBPs) located at the test line of the LFA. Binding to PBPs occurs only with intact (non-hydrolyzed) β-lactams (Fig. 1(b)(i)), resulting in accumulation of the antibiotic-functionalized AuNPs at the PBP test line (red line = negative result , Fig. 1(c)). When exposed to a sample containing active β-lactamases, the hydrolyzed antibiotic-functionalized nanoparticles (Fig. 1(b)(ii)) no longer bind to the PBPs, preventing capture of nanoparticles at the test line (positive test, Fig. 1(c)). This LFA was used to detect a series of five recombinant β-lactamases in phosphate buffer saline (PBS) and in a complex biological media, namely undiluted urine collected from healthy volunteers who were not taking antibiotics. Further investigations were carried out to assess the compatibility of the antibiotic-functionalized AuNPs with microbiological samples, such as culture media and bacterial periplasmic extractions.

## Materials and methods

### AuNP synthesis

**Fig. 2:**
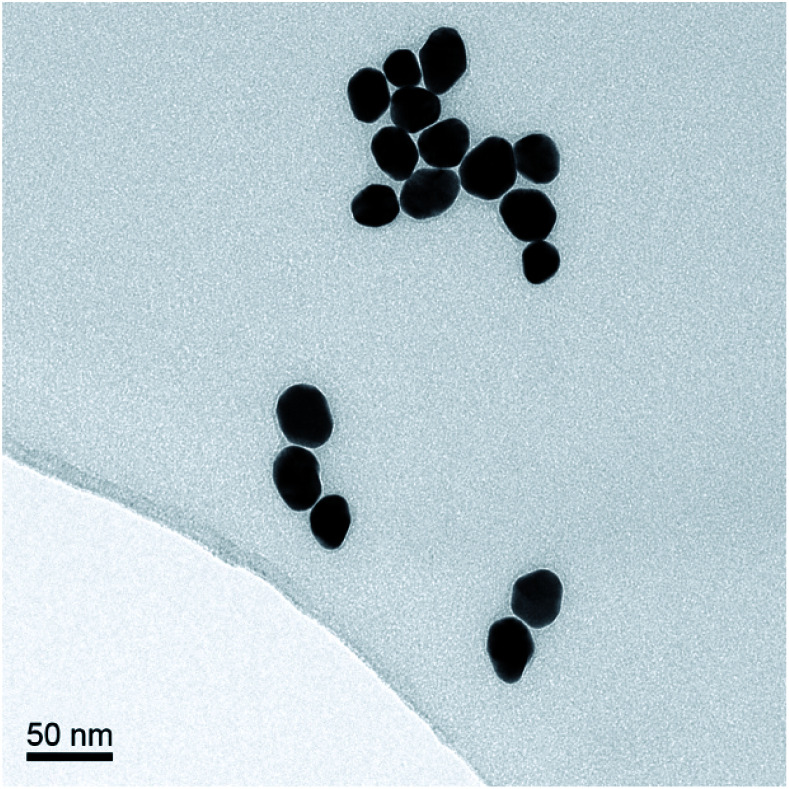
TEM image of AuNPs prepared *via* citrate method.

The AuNPs were synthesized according to the Turkevich method, using sodium citrate to reduce HAuCl_4_.^17^ The average size of the AuNPs was determined by transmission electron microscopy (TEM), Fig. 2. The optical density (OD) was determined using UV/Vis spectrophotometry (Shimadzu UV-1800). The synthesized AuNPs were typically 20 nm in diameter with an extinction coefficient of 9.21 × 10^8^ M^−1^ cm^−1^.

### Synthesis of cephalexin-NHS

**Fig. 3:**
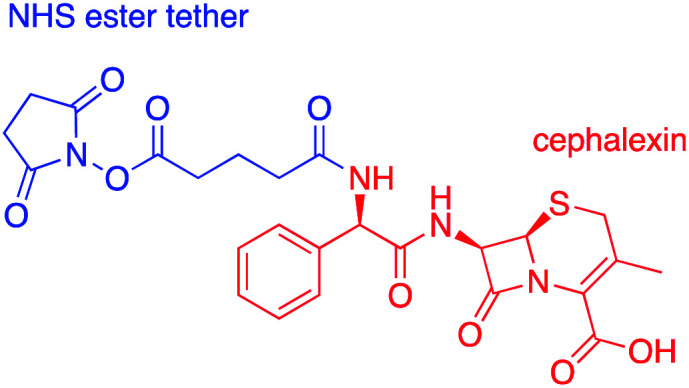
Chemical structure of cephalexin-NHS.

Cephalexin with an *N*-hydroxy succinimidyl (NHS) ester tether (Fig. 3) ((6*R*,7*R*)-7-[(2*R*)-2-{5-[(2,5-dioxopyrrolidin-1-yl)oxy]-5-oxopentanamido}-2-phenylacetamido]-3-methyl-8-oxo-5-thia-1-azabicyclo[4.2.0]oct-2-ene-2-carboxylic acid) was prepared as described previously.^18^

### Functionalization of AuNPs

BSA-AuNPs were prepared as follows: to 1 mL of citrate-stabilized AuNPs (OD 1, measured by absorption at the *λ*_max_) was added 150 μL borate buffer (stock concentration 15 mM, pH 9) followed by 150 μL of bovine serum albumin (BSA) (3% w/v stock in deionized water). The mixture was then shaken at room temperature for 10 min using an orbital shaker. Thereafter the sample was centrifuged (17 000 × *g*, 30 min) and the supernatant discarded. The BSA-AuNPs were then washed, first by resuspension in 500 μL of wash buffer (2 mM borate buffer, pH 9, 5% sucrose, 2% glycerol, 0.5% BSA, and 0.01% Tween) then by resuspension in PBS. Finally, the BSA-AuNPs were resuspended in 1 mL of PBS and the OD measured by absorption at the *λ*_max_.

Cephalexin-AuNPs were prepared as follows: to a solution of BSA-AuNPs, the required cephalexin-NHS analogue was added (stock concentration 1 mg/100 μL in DMSO) to give a final cephalexin-NHS : AuNP ratio of 130 000 : 1. The mixture was shaken at room temperature for 1 h using an orbital shaker, then stored at 4 °C overnight, after which the sample was centrifuged (17 000 × *g*, 30 min) and the supernatant discarded. The AuNPs were then washed with PBS and the final pellet of cephalexin-AuNPs was re-suspended in PBS at the required concentration. The cephalexin-AuNPs were stored at 4 °C until use, batches were used within 48 hours of preparation. Polarization modulation-infrared reflection–adsorption spectroscopy (PM-IRRAS) was used to characterize the surface chemistry used in the preparation of cephalexin-AuNPs (ESI, Fig. S2†).

Streptavidin-AuNPs were prepared as follows: to 1 mL of citrate-stabilized AuNPs (OD 1) was added 150 μL borate buffer (stock concentration 15 mM, pH 9) followed by 150 μL of streptavidin in PBS (50 mg mL^−1^). The mixture was then shaken at room temperature for 10 min using an orbital shaker. Thereafter the sample was centrifuged (17 000 × *g*, 30 min) and the supernatant discarded. The streptavidin-AuNPs were then washed by resuspension in 500 μL of wash buffer (2 mM borate buffer, pH 9, 5% sucrose, 2% glycerol, 0.5% BSA, and 0.01% Tween). The streptavidin-AuNPs were finally washed again with PBS and resuspended in 1 mL of PBS. The OD was measured by absorption at the *λ*_max_.

### Penicillin-binding protein 3

PBP3 (recombinant, *Escherichia coli*) was prepared as described previously.^12^ Briefly, a truncated version of the *fts*I gene that encoded residues 44 to 588, comprising the known soluble domain of FtsI, was amplified from *E. coli* BW25113 cells using whole-cell PCR. The resulting DNA fragment was inserted into the vector pBADnLIC2005,^19^ introducing an *N*-terminal deca-histidine tag when expressed. The PBP3 was purified using a HisTrap HF column (GE Healthcare) using a refolding protocol (2 M guanidine HCl, 50 mM KPi (pH 7.8), 200 mM NaCl, 20% glycerol, and 20 mM imidazole) to remove any bound ligands and then eluted using the elution buffer (50 mM KPi (pH 7.8), 200 mM NaCl, 20% glycerol, and 500 mM imidazole). The protein was then buffer-exchanged to potassium phosphate buffer (KPi) (50 mM, 200 mM NaCl, pH 7.8), using a HisTrap Desalting (GE Healthcare) column. Stock solutions were diluted to the required concentration using PBS. For spotting of PBP3 onto the nitrocellulose membrane, the NaCl concentration of the PBS was increased to 320 mM.

### β-lactamases

Initial enzyme activity testing was carried out using a blend of recombinant β-lactamase proteins, expressed in *E. coli*, purchased from Sigma-Aldrich (L7920) and used without further purification. The lyophilized powder was dissolved in 50 mM KPi (pH 7) and aliquoted into 1 mL aliquots, each containing 40–70 IU β-lactamase I and 6–10 IU β-lactamase II. The enzyme stock solutions were stored at −80 °C. Experiments to test β-lactam hydrolysis by β-lactamases used 5× dilution of the stock enzyme solution. Quartz crystal microbalance with dissipation (QCM-D) experiments employed drug-functionalized surfaces that were pre-incubated in a stock solution of the β-lactamase blend for 16 h at 37 °C.

β-lactamase concentration studies were carried out using purified recombinant proteins. CTX-M-1 and AmpC were purchased from Abcam plc., Cambridge. TEM-1, CTX-M-15, and NDM-1 were expressed in *E. coli* and purified by His-trap.^18^ All β-lactamase solutions were prepared at the desired concentration in the required test media (PBS or urine).

### QCM-D experiments

#### QCM-D sensor functionalization

QCM-D sensors were cleaned by UV–ozone treatment (10 min), followed by sonication in a 2% Hellmanex III solution (Hellma Analytics, Müllheim, Germany) (10 min) and then sonication in Milli-Q water (2 × 10 min), followed by UV–ozone treatment (30 min). Cleaned samples were functionalized according to the type of surface required.

#### BSA-cephalexin surface

Cleaned gold sensors were loaded into the QCM-D instrument and functionalized in flow using the following solutions: (i) BSA (2 mg in 6 mL PBS) and (ii) cephalexin-NHS (3 mg in 6 mL PBS).

#### PBP3 immobilization by His-trap

Cleaned silicon dioxide sensors were functionalized with 3-mercaptopropyltriethoxysilane (MPTES) by immersion in a 4% v/v MPTES/isopropyl alcohol (IPA) solution for 24 h to form a thiol-terminated self-assembled monolayer. Sensors were then rinsed in IPA and dried with N_2_ gas. An IDA-maleimide analogue (see ESI† – compound 4, 2-[*N*-(carboxymethyl)-6-(2,5-dioxo-2,5-dihydro-1*H*-pyrrol-1-yl)hexanamido]acetic acid) was used as the Ni^2+^ chelating agent. Sensors were functionalized with this analogue by immersion in a 0.05% w/v solution of compound 4 in PBS for 24 h. Functionalized sensors were finally rinsed in Milli-Q water and dried with N_2_ gas, before being loaded into the QCM-D flow modules.

#### QCM-D running conditions

Once each sensor was installed into the flow modules of the Q-Sense E4 system, each chamber was filled with Milli-Q water at a flow rate of 100 μL min^−1^ controlled by a four-channel peristaltic pump. To achieve a stable baseline, the initial running solution (Milli-Q water or PBS, see QCM-D results of specific experiments) was left to flow through the modules at 50 μL min^−1^ until the drift in resonant frequency was <±1 Hz over 10 min. For all experiments, the temperature of the modules was maintained at 20 °C (standard deviation 5 × 10^−3^ °C) and the flow rate was kept constant at 50 μL min^−1^. A wash solution (see QCM-D results for solutions used) was allowed to flow over the sensor surface between sample injections until a stable level was achieved, corresponding to a drift in frequency <±1 Hz over 10 min. QCM-D spectra showing both the frequency and dissipation shifts for the seventh overtone of each experiment are provided in the ESI.† Surface concentrations of proteins were calculated using the Sauerbrey equation. We note, the Sauerbrey model assumes a thin, rigid, and uniform layer, and is therefore used here to only provide an estimate of surface concentration.

### LFA materials and construction

Each LFA strip contained four overlapping pads placed on a backing card: sample pad (CFSP203000), conjugate pad (GFCP103000), membrane (HF090MC100), and absorption pad (CFSP203000). Each component overlapped by 2 mm and was adhered to a backing card. Once constructed, PBP3 (in PBS with 320 mM NaCl) was pipetted onto the membrane to form the test spot (stock concentration 750 nM PBP3, 2 μL per strip). The LFA strips were then allowed to dry at room temperature before blocking the membrane by flowing through a solution of BSA in PBS (50 μg mL^−1^). The LFAs were again dried, then cut into 6 mm wide strips and stored in an air tight container at 4 °C.

For LFAs with a control spot, biotinylated-BSA was pipetted to the control region of the membrane (stock concentration 100 μg mL^−1^ in PBS, 0.5 μL per strip) 3 mm above the PBP3 test region.

### LFA running protocol

Prior to testing, LFAs were allowed to return to room temperature and acclimatize to the ambient conditions for 30 min. Unless stated otherwise, samples were assayed as follows: 10 μL cephalexin-AuNPs (1 nM) was added to 10 μL of the test sample and mixed by pipette. The mixture was then incubated at 37 °C for 1 h before adding to the LFA strip. Running buffer (PBS, pH 7.4, 1 mM ETDA, 1% v/v Triton™ X-100) was then added until the membrane was clear in the non-binding areas. The LFA results were then recorded and documented by photograph. Test strips were run in duplicate, alongside strips run with control samples of cephalexin-AuNPs incubated in the test media to assess media effects.

### Urine samples

An “average” human urine sample was used, as described previously.^12^ Collection and handling of urine samples was performed following procedures pre-approved by the University of York's Department of Biology Ethics Committee and to comply with the UK Concordat to Support Research Integrity (2019). Informed consent was collected from all sample donors. Urine samples were collected anonymously from 13 healthy adults that had not shown symptoms of infection or had taken antibiotics within 1 month prior to the sample collection. Participants were recruited from within the University of York. Samples were filter-sterilized using 0.22 μm syringe filters to remove any cells and then pooled to form an “average” human urine, which was aliquoted and stored in a −80 °C freezer. The pH of the resulting urine was measured to be pH 6.7. Control experiments did not detect any β-lactamases within the urine stock.

## Results and discussion

### Antibiotic-functionalized AuNPs

AuNPs (synthesized *via* the Turkevich method,^17^ and characterized by UV-Vis spectrophotometry and TEM, details provided in the Experimental section and the ES1†) were first functionalized with BSA to produce a protein coating that was found to preserve hydrophilicity (optimization of AuNP surface chemistry is provided in the ESI†). Furthermore, this layer was designed to act as a barrier to reduce biofouling (non-specific adsorption of proteins) of the AuNPs when used for detection in biological samples, such as urine. The BSA-AuNPs were then further functionalized with probe antibiotics using an NHS ester analogue of cephalexin (chemical structure shown in Fig. 3), which reacts with the lysine residues of BSA to attach the antibiotic *via* amide bonds, Fig. 1(b)(i). Optimization of the concentration of cephalexin-NHS was required as at high concentrations the antibiotic-functionalized AuNPs became too hydrophobic, but at low concentrations there was insufficient binding of the AuNPs to the test line of the LFA. Using QCM-D data, it was calculated that in saturated surfaces functionalized with BSA-cephalexin, there are 6050 cephalexin molecules bound per 20 nm AuNP (ESI Table S3†). Stability tests found that the optimized cephalexin-BSA-AuNPs were stable in up to 100% urine (ESI Fig. S4 and Table S2†).

### Antibiotic-functionalized AuNPs binding experiments

QCM-D was used to ascertain whether cephalexin molecules bound to a surface *via* a BSA layer were able to bind to PBP3 and whether β-lactamases could access and hydrolyze the immobilized cephalexin. For clarity, only the frequency data of key steps in the QCM-D experiments are discussed herein, with the full experimental data, including dissipation data, provided in the ESI.†

**Fig. 4:**
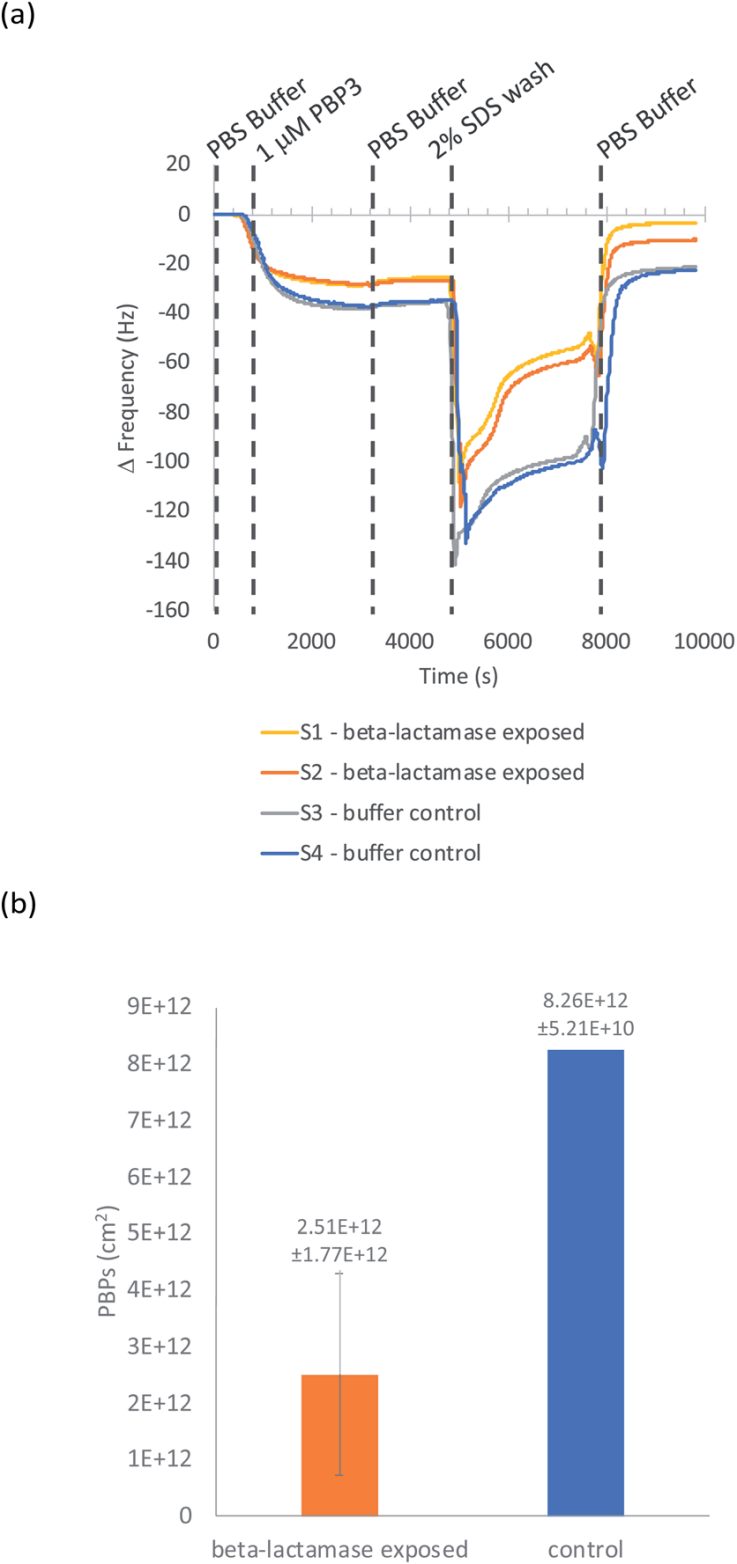
(a) QCM-D experiment of PBP3 binding to a BSA-cephalexin surface; (b) the number of PBPs bound after the final wash for +/− β-lactamase. Data reported as mean value with ± standard deviation error bars.

Previously, we demonstrated that cephalexin attached to a thiol surface *via* a polyethylene glycol (PEG) linker or a short alkane tether were able to react with PBP3 and β-lactamases.^16^ To investigate the BSA-cephalexin surface chemistry developed for AuNPs functionalization (Fig. 1(b)(i)), Au-coated QCM-D sensors were first functionalized with BSA followed by attachment of cephalexin using the NHS ester analogue (Fig. 3). The drug-covered surface was subsequently challenged with a solution of PBP3 followed by a washing step (Fig. 4). The average magnitude of the resulting frequency shift (−23 Hz) suggested that approximately 8.3 × 10^12^ PBPs per cm^2^ were bound to the β-lactam antibiotics on the surface (S3 and S4†). This surface density is 83% of the density previously reported for a monolayer of PBP3 (1 × 10^13^ proteins per cm^2^).^12^ We also note, the PBPs remained bound to the surface following washing with 2% sodium dodecyl sulfate (SDS) confirming that the antibiotics immobilized using this surface chemistry were able to covalently bind with the PBPs. In a parallel experiment, two of the cephalexin-functionalized sensors were pre-exposed to a solution containing a blend of β-lactamases before introducing the PBP3 solution (S1 and S2†). In order for the LFA to detect active β-lactamases, the enzymes must be able to hydrolyze the surface-bound antibiotics, thereby preventing binding between cephalexin and PBP. As shown in Fig. 4, the amount of PBPs binding was reduced by 60% following exposure of the cephalexin surface to β-lactamase, thus demonstrating that a significant number of the BSA-bound antibiotics were hydrolyzed and therefore unable to bind the PBPs.

**Fig. 5:**
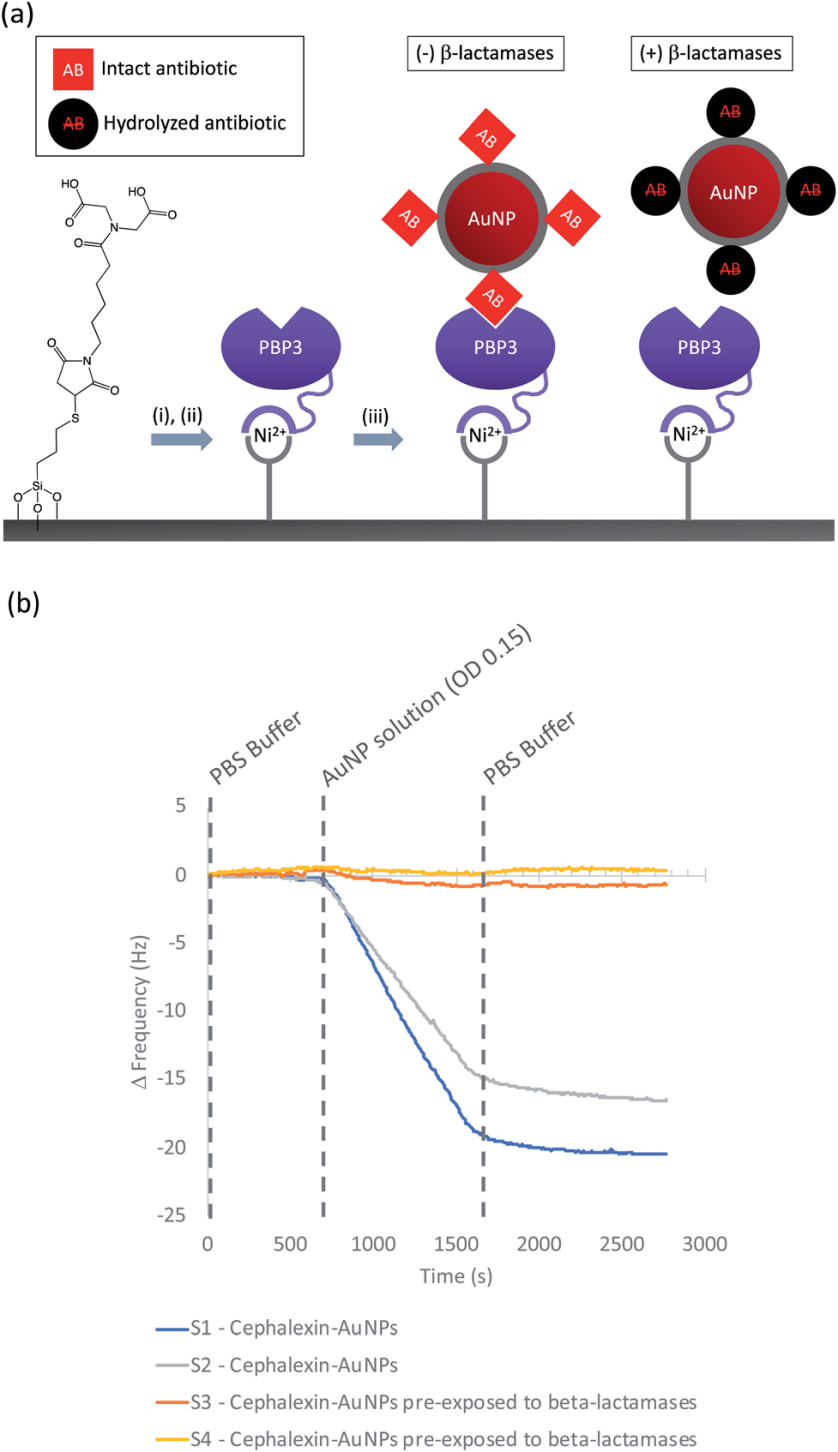
(a) Immobilization steps used in QCM-D experiment to replicate the LFA conditions: (i) NiSO_4_, (ii) PBP3 immobilization (iii) Antibiotic-functionalized AuNPs-only binding if antibiotics are intact; (b) QCM-D experiment with immobilized PBP3: S1/S2 confirmed binding of intact antibiotic-functionalized AuNPs; S3/S4 show no binding to PBP3 when the antibiotic-functionalized AuNPs were pre-exposed to β-lactamases.

Having demonstrated the immobilization chemistry on planar surfaces, further QCM-D experiments were performed to investigate cephalexin-PBP interactions using the antibiotic-functionalized AuNPs. In order to immobilize the PBP3 onto the sensor surface a His-tag strategy was used as shown in Fig. 5(a): SiO_2_ sensors were first functionalized with MPTES to provide a thiol handle for the attachment of a Ni^2+^ binding motif using maleimide conjugation. The functionalized sensors were then loaded into the QCM-D flow modules and a NiSO_4_ solution was used to load the surface with Ni^2+^ followed by a buffer washing step to remove excess reagents (ESI Fig. S6†). The sensor surfaces were then exposed to a solution of PBP3 with an *N*-terminal deca-His-tag followed by another washing step to remove non-specifically bound proteins. Once functionalized with PBP3, the surfaces were challenged with one of two samples: (i) antibiotic-functionalized AuNPs and (ii) antibiotic-functionalized AuNPs in which the cephalexin was hydrolyzed by pre-exposure to a solution of β-lactamases. As shown in Fig. 5(b), we observed significant binding between the antibiotic-functionalized AuNPs and the surface immobilized PBP3 (S1 and S2†), whereas the AuNPs that were pre-incubated with β-lactamases did not bind to the PBP3 surface (S3 and S4†).

These experiments confirm that the cephalexin-AuNPs can be used to probe for/detect β-lactamase activity, with formation of the PBP-cephalexin-AuNPs complex indicating the absence of β-lactamase activity.

### LFA optimization

A total of 15 membrane types were trialed for use in the LFA, and HF090MC100 was identified as the optimal membrane. See the ESI (Table S4†) for the full list of the membranes investigated.

**Fig. 6:**
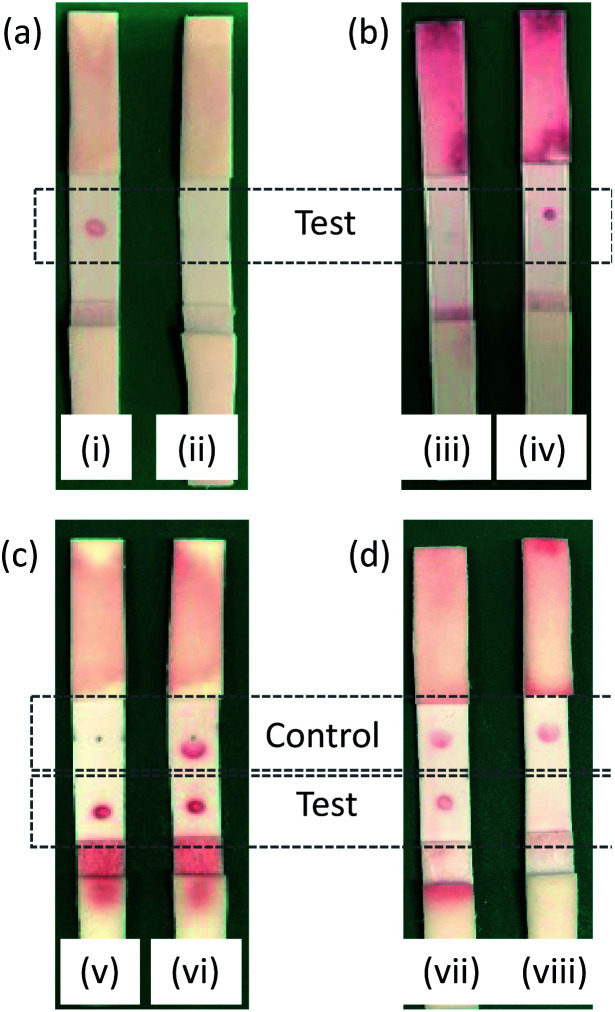
LFA test results, red color due to AuNPs present: (a) Specificity of (i) cephalexin-AuNPs *vs.* (ii) BSA-AuNPs; (b) proof of concept in detecting β-lactamases activity using antibiotic-functionalized AuNPs: (iii) preincubated with β-lactamases and (iv) no β-lactamases; (c) compatibility of antibiotic-functionalized AuNPs with a streptavidin/biotin control: (v) antibiotic-functionalized AuNPs only *vs.* (vi) antibiotic-functionalized AuNPs mixed with streptavidin-AuNPs; (d) β-lactamase tests using a mixture of antibiotic-functionalized AuNPs and streptavidin-AuNPs: (vii) mixed AuNPs, no β-lactamases and (viii) mixed AuNPs, preincubated with β-lactamases.

Test strips were prepared containing PBP3 as the test spot. Initial tests employed two different species of AuNP. The first were AuNPs functionalized with BSA, designed to test the selectivity of the test spot and assess non-specific binding. The second were AuNPs functionalized with BSA-cephalexin, which would bind to the PBP3 of the test spot provided the cephalexin molecules were active. Preliminary tests revealed issues with non-specific binding to the test spot, which was rectified by the optimization of the running buffer to PBS with 1 mM EDTA and 1% v/v Triton™ X-100. All subsequent experiments employed this optimized buffer. It was successfully shown that the cephalexin-AuNPs were selective for PBP3 and with no detectable background signal due to non-specific binding, Fig. 6(a). Furthermore, after exposing the cephalexin-AuNPs to a blend of recombinant β-lactamases for 5 h, the AuNPs were no longer able to bind to the test spot (Fig. 6(b)). This demonstrates the principle of using the cephalexin-AuNPs in an LFA to detect β-lactamase activity; with a visible red spot at the PBP test region indicating no β-lactamases present or lack of β-lactamase activity, whereas the absence of a spot would indicate that active β-lactamases are present (Fig. 6(b)).

The concentration of PBP3 within the test spot was then optimized to ensure sufficient binding of AuNPs for a clearly visible colored spot appearing in the test region. It was also critical to maintain the flow of the AuNPs through the membrane that could otherwise produce false readings. An initial screen of 2 μL spots at 10 μM, 1 μM, 100 nM, 10 nM, and 1 nM of PBP3 in PBS found that 1 μM was optimal (ESI Fig. S7†). A second screen over the narrower range of 2 μM–125 nM, identified that non-specific binding was occurring at the higher concentrations; ultimately, 750 nM was identified as the optimal concentration. During initial trials, it was noted that the test spot appeared in a ring shape with a clear region in the center (*e.g.*Fig. 6(i)), indicating that the protein was not evenly immobilized onto the membrane during the preparation of the test strips. This was remedied by increasing the NaCl concentration of the PBP3 solution used when spotting the protein onto the membrane (from 137 mM to 320 mM).

Optimized LFAs were prepared and used to optimize the concentration of cephalexin-NHS used in the preparation of the antibiotic-functionalized AuNPs. By testing across a concentration gradient, it was determined that a final cephalexin-NHS : AuNP ratio of 130 000 : 1 was optimal. Due to the unstable nature of NHS esters under aqueous conditions, a large proportion of the tethered antibiotics are likely to be hydrolyzed in the PBS buffer before reacting with the BSA lysine residues. To minimize this loss, a stock solution of cephalexin-NHS in DMSO at 1 mg 100 μL^−1^ was used, with the addition of the required volume to the PBS solution of BSA-AuNP to give the required ratio.

### Limit of detection *in vitro*

The sensitivity of the LFA was investigated using five selected recombinant β-lactamases in buffer. The β-lactamase family of enzymes is vast, with over 2500 unique proteins identified.^20^ β-lactamases can be categorized into subsets (class A, B, C, or D), based on their protein sequence. TEM-1 was included as this class A β-lactamase is one of the most common β-lactamases found in Gram-negative bacteria.^21^ AmpC is a class C β-lactamase known to confer resistance to cephalosporins including cephalexin, the antibiotic used in the preparation of the antibiotic-functionalized AuNPs.^20^ CTX-M-1 and CTX-M-15 are ESBLs from class A; these plasmid-encoded enzymes^22^ are associated with multi-drug resistant infections.^23,24^ The class B metallo-β-lactamase NDM-1 was also included, known to hydrolyze penicillins, cephalosporins, and even last resort carbapenems by a mechanism catalyzed by one or two zinc ions present in the active site.^25^

Each of the five selected β-lactamases was tested across a range of concentrations, in 10× incremental dilutions. The antibiotic-functionalized AuNPs were incubated in these prepared solutions for 1 h at 37 °C before being added to the LFA strips. A screen of three different temperatures (20 °C, 37 °C, and 50 °C) identified 37 °C to be the optimal temperature for the incubation step (Table S5†). Details on the concentrations screened are provided in the (Tables S6(a) and (b)†). Ideally, for point-of-care applications the diagnostics should be rapid, thus the detection limit after 1 h incubation with β-lactamases was investigated. The test results and limits of detection (LOD) for each of the selected β-lactamases are shown in Table 1.

**Table 1:** LOD of β-lactamases in buffer and in urine, after 1 h incubation

β-Lactamase	Buffer LOD	Urine LOD
TEM-1	6 μM	6 μM
AmpC	12 μM	12 μM
CTX-M-1	780 nM	7.8 μM
CTX-M-15	60 nM	60 nM
NDM-1	11 nM	110 nM

Further testing was also performed in urine spiked with β-lactamases (Table 1) to investigate the effects of running the diagnostic in a complex media. The LOD was 10-fold less sensitive for the detection of CTX-M-1 and NDM-1 in urine; however, for the three other β-lactamases trialed, the LOD in urine was comparable with the results obtained using buffer. These results demonstrated that the design of the antibiotic-functionalized AuNPs and membrane-blocking protocol were successful in minimizing biofouling. Further control tests confirmed that 2% boric acid, a preservative commonly added to urine samples in a clinical setting, did not affect the LFA. The LFA is also compatible with up to 2.5% v/v blood in urine, suggesting that the antibiotic-functionalized AuNPs would be suitable for detecting in infections presenting with hematuria (ESI Table S7†).

Previous studies have reported that the periplasmic concentrations of β-lactamases can range from 3.7 μM - 1 mM in an *E. coli* cell.^26–28^*E. coli* is the most common pathogen in UTIs, with a urine sample typically containing between 10^4^ to 10^6^ CFU mL^−1^. Using these reported values, it is expected that the β-lactamase concentration in a UTI urine sample will range between 2.4 fM to 66 pM (assuming the β-lactamases are fully lysed from mature bacterial cells with a periplasmic volume of 20% of the total cell volume). To confirm this predicted range, the β-lactamase concentration of cell lysates was determined for two strains of *E. coli*, expressing different β-lactamases, using the nitrocefin assay.^29^ This colorimetric assay is not sufficiently sensitive to detect β-lactamases at the relevant concentrations; however, through linear regression of the correlation of CFU mL^−1^ to β-lactamase concentration (ESI Fig. S11 and S12†), the β-lactamase concentrations of UTI samples with 10^4^ to 10^6^ CFU mL^−1^ of the two selected strains were found to fall within the predicted concentration range of β-lactamases (ESI Table S8†). These results identified that enzyme pre-concentration or bacterial culturing would be necessary for the direct detection of active β-lactamases in a UTI sample. This was confirmed through tests carried out using the LFA with simulated UTI urine samples at 10^6^ CFU mL^−1^ of CTX-M-15 producing *E. coli* (data not shown). We note, the antibiotic-functionalized AuNPs were found to be compatible in microbiologically-important systems such as LB and lysis buffers (specifically, BugBuster® as well as PBS with 500 mM sucrose and 1 mM EDTA, pH 7.4, for periplasmic extractions) allowing the LFA to also be used following bacterial culturing.

The LFA described herein provides proof of concept towards the development of a point-of-care test for active β-lactamases in a patient's sample. To reach the required sensitivity for direct testing for the enzyme activity in urine samples, alternative antibiotic-functionalized nanoparticles (NPs) could be implemented in this LFA platform. In nanodiagnostics, AuNPs used in a colorimetric readout (as in the LFA detailed here) is a common choice; however, this method is reported to have low sensitivity, typically within the μM-pM range.^30^ By using alternative NPs the sensitivity could be significantly improved, such as fluorescent NPs (nM to fM range) or magnetic NPs (pM to aM range). Another approach would be to adapt the LFA using AuNPs to have a more sensitive readout method, as the optical color change read by eye is limited by a higher concentration of AuNPs required to produce the visible red line. For example, it has been shown that using photon counting to quantify the AuNPs binding in an LFA can greatly improve the LOD.^31^ For a recent review on approaches to improving the sensitivity of LFAs, see the perspective by Bishop *et al.*^32^

## Summary and conclusion

Antibiotic-functionalized AuNPs have been successfully developed for the detection of β-lactamases in complex media. Using a surface chemistry strategy that includes a foundation layer of BSA on the AuNPs, produced hydrophilic AuNPs that were resistant to biofouling and allowed for application of these detection tools in biological samples such as urine, LB, and periplasmic extractions. QCM-D studies confirmed that these antibiotic-functionalized AuNPs were recognized by β-lactamases, resulting in the hydrolysis of surface-bound antibiotics. It was also shown that the binding of these antibiotic-functionalized AuNPs to PBP3 can be used to indicate that the antibiotics were intact, thus monitoring for the binding interaction can infer the presence or absence of β-lactamases.

The antibiotic-functionalized AuNPs were successfully applied to an LFA platform as a simple test for the presence of active β-lactamases. The LFA was shown to detect one of the investigated β-lactamases (NDM-1) down to a concentration of 11 nM. With estimated β-lactamase concentrations in UTI urine samples ranging from fM to pM, pre-concentration steps would have to precede sample application to enable detection. Notably, it was found that the test is more sensitive to the enzymes found in difficult-to-treat infections (CTX-M-15 and NDM-1) over the more standard β-lactamases (TEM-1 and AmpC). Investigations using undiluted urine spiked with β-lactamases demonstrated the compatibility of the antibiotic-functionalized AuNPs in detecting β-lactamase activity in complex media. The LFA described herein allows for the detection of β-lactamase activity, thus providing an alternative method of detecting these enzymes, that is complementary to antibody-based testing.

This work provides proof of concept that these antibiotic-functionalized AuNPs can be used to develop biosensors for one of the major contributors towards AMR. By using AuNPs, there is a plethora of technologies in which these novel tools of detection are compatible, which could lead to the development of vital point-of-care diagnostics for bacterial infections.

## Abbreviations

AuNPs: Gold nanoparticles
BSA: Bovine serum albumin
DMSO: Dimethyl sulfoxide
EDTA: Ethylenediaminetetraacetic acid
ESBL: Extended-spectrum β-lactamases
IPA: Isopropyl alcohol
KPi: Potassium phosphate
LB: Luria–Bertani broth
LFA: Lateral flow assay
LOD: Limit of detection
MPTES: 3-Mercaptopropyltriethoxysilane
ND: Not detected
NHS: *N*-Hydroxy succinimidyl
QCM-D: Quartz crystal microbalance with dissipation
PEG: Polyethylene glycol
PBP: Penicillin-binding protein
PBS: Phosphate buffer saline
PM-IRRAS: Polarization modulation-infrared reflection-adsorption spectroscopy
rpm: Rotations per minute
SDS: Sodium dodecyl dulfate
UTI: Urinary tract infection

## Author contributions

Conceptualization, LMM; methodology, LMM and MDS; investigation, LMM and CDS; resources, GHT, SJ, and AKDK.; writing—original draft preparation, LMM; writing—review and editing, LMM, MS, TFK, GHT, SJ, and AKDK. Funding acquisition, LMM and AKDK (Grow MedTech), and TFK, GHT, SJ, and AKDK (EPSRC). All authors have read and agreed to the published version of the manuscript.

## Conflicts of interest

The authors declare that they have no known competing financial interests or personal relationships that could have influenced the work reported in this paper.

## Supplementary Material

NA-004-D1NA00635E-s001
