# Supplementary material for: Antibiotic-functionalized gold nanoparticles for the detection of active β-lactamases
Source: Nanoscale Adv. 2021 Dec 13;4(2):573–81. doi: 10.1039/d1na00635e (PMC9419081; doi:10.1039/d1na00635e)
Supplement: NA-004-D1NA00635E-s001 [file NA-004-D1NA00635E-s001.pdf]

Electronic Supplementary Material (ESI) for Nanoscale Advances.
This journal is © The Royal Society of Chemistry 2021

Supporting Information

Antibiotic-functionalized Gold Nanoparticles for the Detection of Active β-lactamases

Lisa M. Miller,*[1] Matthew D. Simmons,[2] Callum D. Silver,[3] Thomas F. Krauss,[3] Gavin H. Thomas,[4] Steven D. Johnson[2] and Anne-Kathrin Duhme-Klair[1]

[1]Department of Chemistry, [2]Department of Electronic Engineering, [3]Department of Physics, and [4]Department of Biology, University of York, Heslington, York, YO10 5DD, UK.

*lisa.miller@york.ac.uk

1. **Surface Chemistry Optimization**
    1.1. PM-IRRAS Characterization

2. **Gold Nanoparticle Characterization**
    2.1. Gold nanoparticle functionalization
    2.2. Gold nanoparticle stability in urine

3. **QCM-D Proof of Concept Experiments**
    3.1. Preparation of NTA analogue (compound **4**)
    3.2. BSA-cephalexin surface full experiment
        3.2.1. Binding Sites on BSA-cephalexin AuNPs
    3.3. PBP3 surface full experiment

4. **LFA Optimization**
    4.1. Membrane optimization
    4.2. PBP3 spotting in test zone of LFA
    4.3. Biotinylated-BSA spotting in control compatibility studies

5. **LFA Testing**
    5.1. Incubation temperature studies
    5.2. Limit of detection *in vitro*
        5.2.1. *In vitro* results with LFA
        5.2.2. *In vitro* results with CTX-M and CTX-M M

6. **Compatibility with Hematuria**

7. **Correlation of β-lactamase concentration**
    7.1. Nitrocefin assay protocol
    7.2. CFU/mL Determination

1. Surface Chemistry Optimization

Initial batches of antibiotic-functionalized AuNPs were prepared using a lipoic acid analogue of cephalexin, Fig. S1 (i). The synthesis of this analogue was described previously in our investigation of tethered cephalexin analogues.[18] Disulfides are a common functional handle used in the functionalization of gold surfaces; however, it was found that the use of this tethered antibiotic analogue produced hydrophobic AuNPs that were difficult to handle and prone to aggregation. A common solution to this issue is the introduction of a polyethylene glycol (PEG) chain, to produce a more flexible and hydrophilic tether. However, AuNPs functionalized using a PEG linker terminating with the antibiotic (Fig. S1 (ii)) were found to readily aggregate in aqueous solutions, thus the alternative BSA-based strategy was taken (Fig. S1 (iii)).

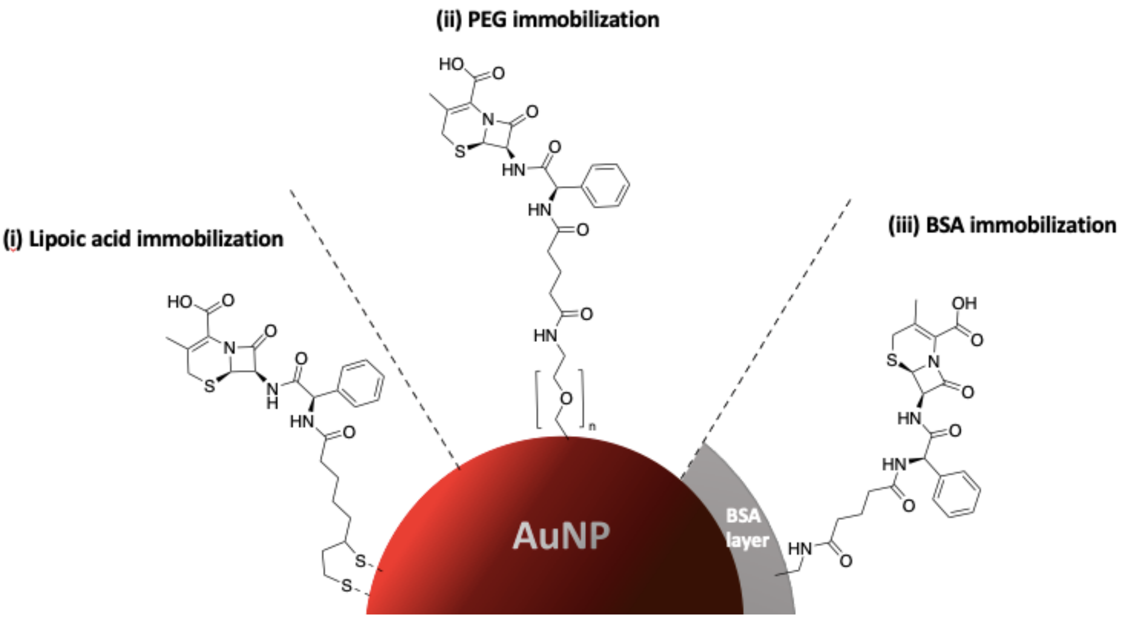

Fig. S1: Optimization of antibiotic-functionalized AuNPs surface chemistry: (i) lipoic acid analogue of cephalexin attached directly to AuNP; (ii) NHS-ester analogue of cephalexin conjugated to amine-PEG-functionalized AuNP, average M_n 5,000; (iii) NHS-ester analogue of cephalexin conjugated to BSA-functionalized AuNP.

1.1. PM-IRRAS Characterization

PM-IRRAS was used to characterize the two steps of the surface functionalization: (i) BSA-functionalized and (ii) BSA-cephalexin-functionalized. The spectra are provided in Fig. S2.

PM-IRRAS spectra were acquired using a Bruker Vertex 70 spectrometer coupled with a PMA50 polarization modulation unit (Hinds Instruments, Oregon, USA). Gold wafers for PM-IRRAS were cleaned by immersion in piranha solution (H_2SO_4/H_2O_2 70:30) for 10 min, followed by sonication in water and ethanol for 10 min each. Samples were functionalized with BSA by immersion in a 0.33 mg/mL BSA in PBS solution for 30 min followed by a washing step using PBS containing 0.1% Tween® 20. Subsequent functionalization with cephalexin was carried out by immersion of the BSA-functionalized sample in a 0.5 mg/mL NHS-cephalexin in PBS solution overnight followed by a washing step using PBS.

Functionalized samples were then loaded into the PM-IRRAS spectrometer. The incident angle was set at 80° with a 4 cm^{-1} spectral resolution, while the PEM controller operated at 2000 cm^{-1}. Measurement time was 10 min, collecting 600 scans.

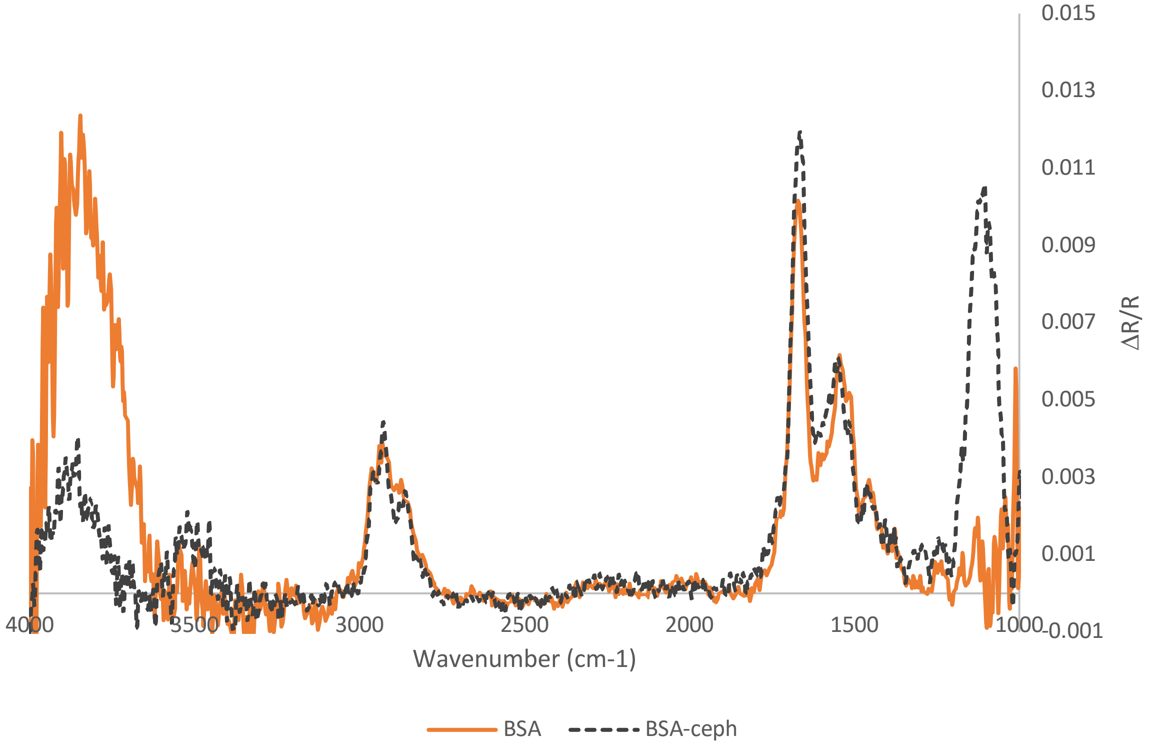

Fig. S2: PM-IRRAS spectra of the BSA-functionalized surface (orange, solid) and BSA-cephalexin-functionalized surface (grey, dashed).

2. Gold nanoparticle characterization

2.1. Gold nanoparticle functionalization

The AuNPs were synthesized according to the Turkevich method, using sodium citrate to reduce $HAuCl_4$.[17] The average size of the AuNPs was characterized by transmission electron microscopy (TEM). The OD were determined using UV/Vis spectrophotometry (Shimadzu UV-1800). The synthesized AuNPs were typically 20 nm in diameter with an extinction coefficient of $9.21 \times 10^8\ M^{-1}\ cm^{-1}$.

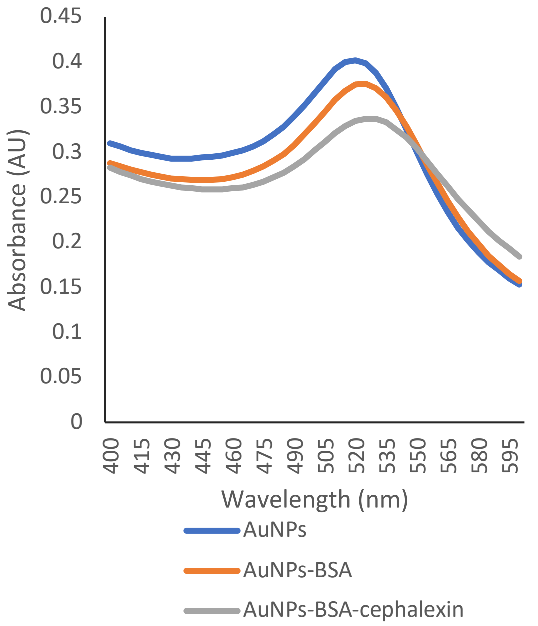

Fig. S3: UV-vis spectrum of AuNPs at each stage of surface functionalization.

Table S1. UV-vis analysis of AuNPs

Sample	λmax (nm)	Absorbance (AU)
AuNPs	520	0.402
AuNPs-BSA	525	0.376
AuNPs-BSA-cephalexin	525-530	0.337

2.2. Gold nanoparticle stability in urine

Six solutions were prepared with increasing amounts of urine in PBS buffer from 0% urine to 100% urine, as detailed in Table S2. The absorbance from 400-600 nm for each solution was measured to account for any background absorbance from the urine. Pre-prepared cephalexin-BSA-AuNPS (cephAuNPs) were suspended in each solution, incubated at room temperature for 10 min, and then the absorbance from 400-600 nm was measured. The difference in absorbance (sample – background) was plotted, as shown in Fig. S4. The λmax and absorbances of each sample are summarized in Table S2. These stability tests found that the optimized cephalexin-BSA-AuNPs were stable in up to 100% urine.

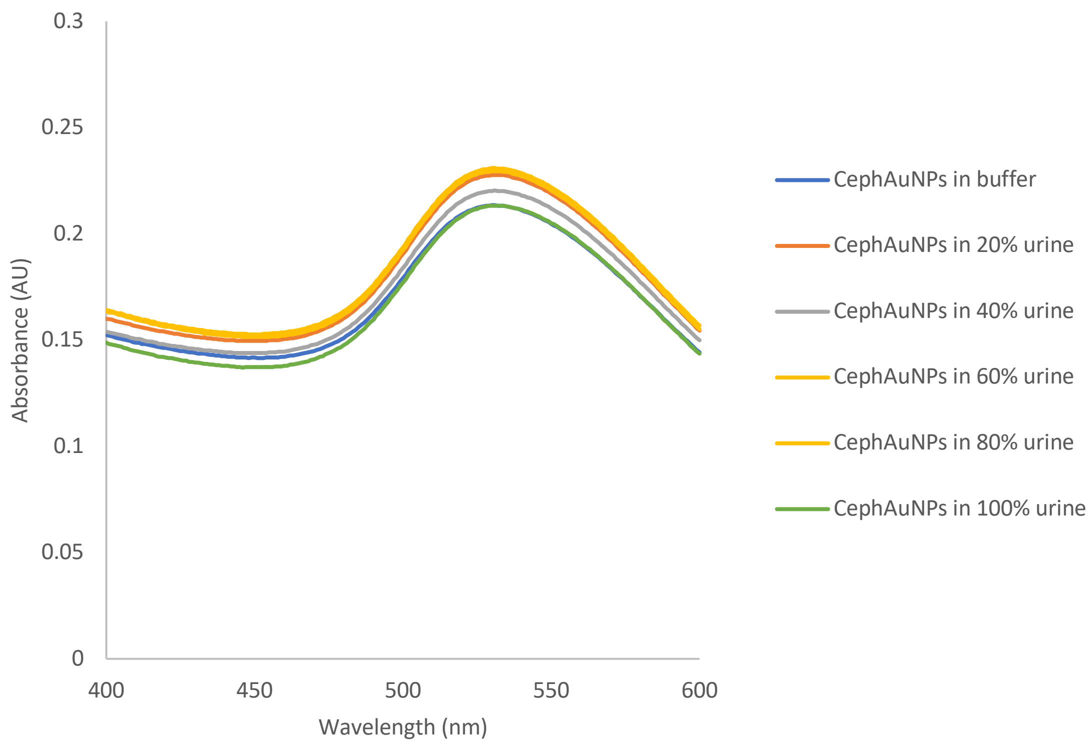

Fig. S4: UV-vis spectrum of antibiotic-functionalized AuNPs in urine.

Table S2. UV-vis analysis of cephalexin-BSA-AuNPs in urine

Media	λmax (nm)	Absorbance (AU)
Buffer	530	0.284
20% urine	530	0.274
40% urine	530	0.261
60% urine	525	0.239
80% urine	525	0.237
100% urine	520	0.213

3. QCM-D proof of concept experiments

3.1. Preparation of NTA analogue (compound 4)

Materials: Di-*tert*-butyl iminodiacetate (**2**) was purchased from TCI. 6-Maleimidohexanic acid (**1**) was purchased from Alfa Aesar. HATU was purchased from Fluorochem. TFA was purchased from Fisher. All solvents were purchased from Fisher. All reagents and solvents were used as supplied.

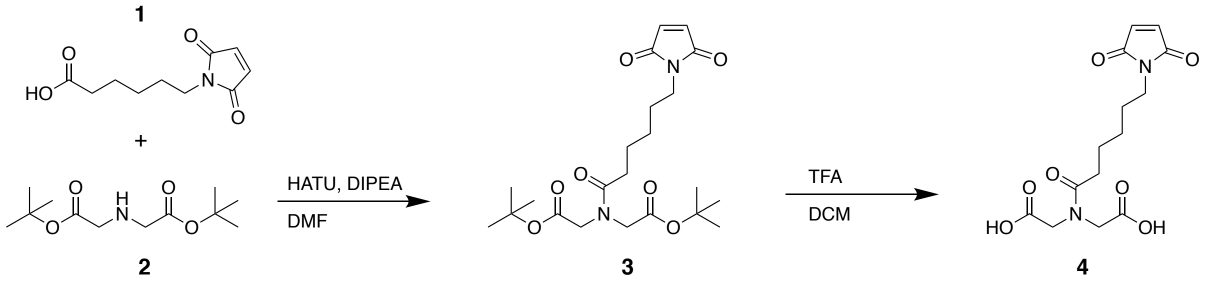

Step 1: Synthesis of Compound **3**: *tert*-butyl 2-{*N*-[2-(tert-butoxy)-2-oxoethyl]-6-(2,5-dioxo-2,5-dihydro-1H-pyrrol-1-yl)hexanamido}acetate

6-Maleimidohexanic acid (**1**) (100 mg, 0.474 mmol, 1 equiv.) was dissolved in DMF (5 mL, 0.1 M) followed by the addition of HATU (216 mg, 0.569 mmol, 1.2 equiv.) and DIPEA (165 μL, 0.948 mmol, 2 equiv.). The resultant solution was stirred for 10 minutes before adding di-tert-butyl iminodiacetate (**3**) (110 mg, 0.450 mmol, 0.95 equiv.) and stirring for a further 16 hours at room temperature. The reaction mixture was then concentrated under reduced pressure and the residue dissolved in EtOAc (15 mL). The EtOAc solution was then washed with 0.01 M HCl (15 mL) and brine (15 mL). The organic layer was then collected, dried using Mg_2SO_4, and concentrated to a brown oil. The crude oil was then purified by column chromatography, eluting with 20-40% EtOAc/petroleum ether, to afford the desired compound **3** as a colorless gum (137 mg, 69% yield).

^{1}H NMR (400 MHz, Chloroform-*d*) δ 6.67 (s, 2H), 4.05 (s, 2H), 3.99 (s, 2H), 3.51 (t, *J* = 7.3 Hz, 2H), 2.31 – 2.23 (m, 2H), 1.72 – 1.57 (m, 4H), 1.47 (s, 9H), 1.45 (s, 9H), 1.38 – 1.29 (m, 2H). **HRMS**: exact mass calculated for [M+Na$^+$] ($C_{22}H_{34}N_2NaO_7$) required *m/z* 461.2258, measured *m/z* 461.2258.

Step 2: Synthesis of Compound **4**: 2-[*N*-(carboxymethyl)-6-(2,5-dioxo-2,5-dihydro-1H-pyrrol-1-yl)hexanamido]acetic acid

To a solution of *tert*-butyl 2-{*N*-[2-(tert-butoxy)-2-oxoethyl]-6-(2,5-dioxo-2,5-dihydro-1H-pyrrol-1-yl)hexanamido}acetate (**3**) (130 mg, 0.297 mmol, 1 equiv.) in DCM (3 mL, 0.1 M) was added TFA (200 μL) and the reaction mixture was stirred at room temperature overnight. The reaction was then concentrated under reduced pressure and the residue triturated in diethyl ether to afford the desired compound **4** as a white solid (88 mg, 91% yield).

^{1}H NMR (400 MHz, DMSO-*d*6) δ 7.00 (s, 2H), 4.14 (s, 2H), 3.94 (s, 2H), 3.36 (t, *J* = 7.1 Hz, 2H), 2.21 (t, *J* = 7.4 Hz, 2H), 1.53 – 1.39 (m, 4H), 1.25 – 1.14 (m, 2H). **^{13}C NMR** (101 MHz, DMSO-D_6) δ 172.8, 171.2, 170.8, 134.5, 49.9, 48.2, 37.0, 31.6, 27.9, 25.8, 24.0. **HRMS**: exact mass calculated for [M+H$^+$] ($C_{14}H_{19}N_2O_7$) required *m/z* 327.1187, measured *m/z* 327.1188. **IR** (neat): 2939, 1729, 1698, 1609, 1475, 1438, 1410, 1371, 1211,1181, 1141 cm^{-1}.

3.2. BSA-cephalexin surface full experiment

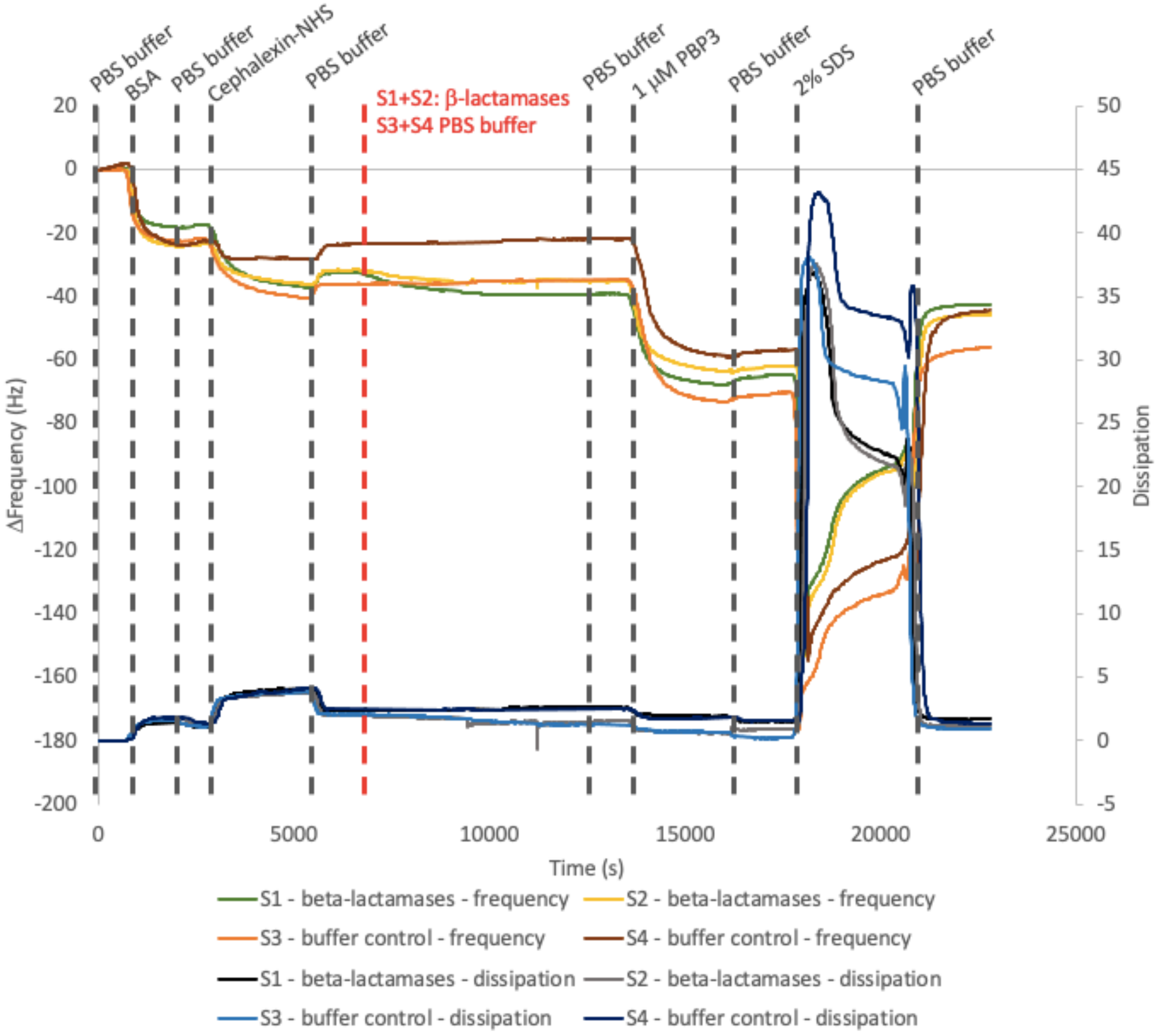

Fig. S5: QCM-D experiment with immobilization of cephalexin attached to a BSA layer and introduction of PBP3 to determine the difference between surfaces exposed to β-lactamase (S1 and S2) vs control surfaces with no β-lactamases (S3 and S4).

3.2.1. Binding Sites on BSA-cephalexin AuNPs

Surface concentrations of cephalexin molecules was calculated from the average of the four QCM-D experiments in Fig. S5 using the Sauerbrey equation. We note, the Sauerbrey model assumes a thin, rigid, and uniform layer, and is therefore used here to only provide an estimate of surface concentration.

Table S3. Calculation of binding sites per BSA-cephalexin functionalized AuNP

**Average frequency shift (Hz)**	-9.64168514
**Change in mass (g)[a]**	5.67158E-07
**Molecules per cm^2 [b]**	4.80202E+14
**Binding sites per AuNP [c]**	6050

[a]Calculated using the Sauerbrey equation; [b]QCM-D sensor surface area 1.593 cm^2; [c]20 nm AuNPs surface area of 1260 nm^2

3.3. PBP3 surface full experiment

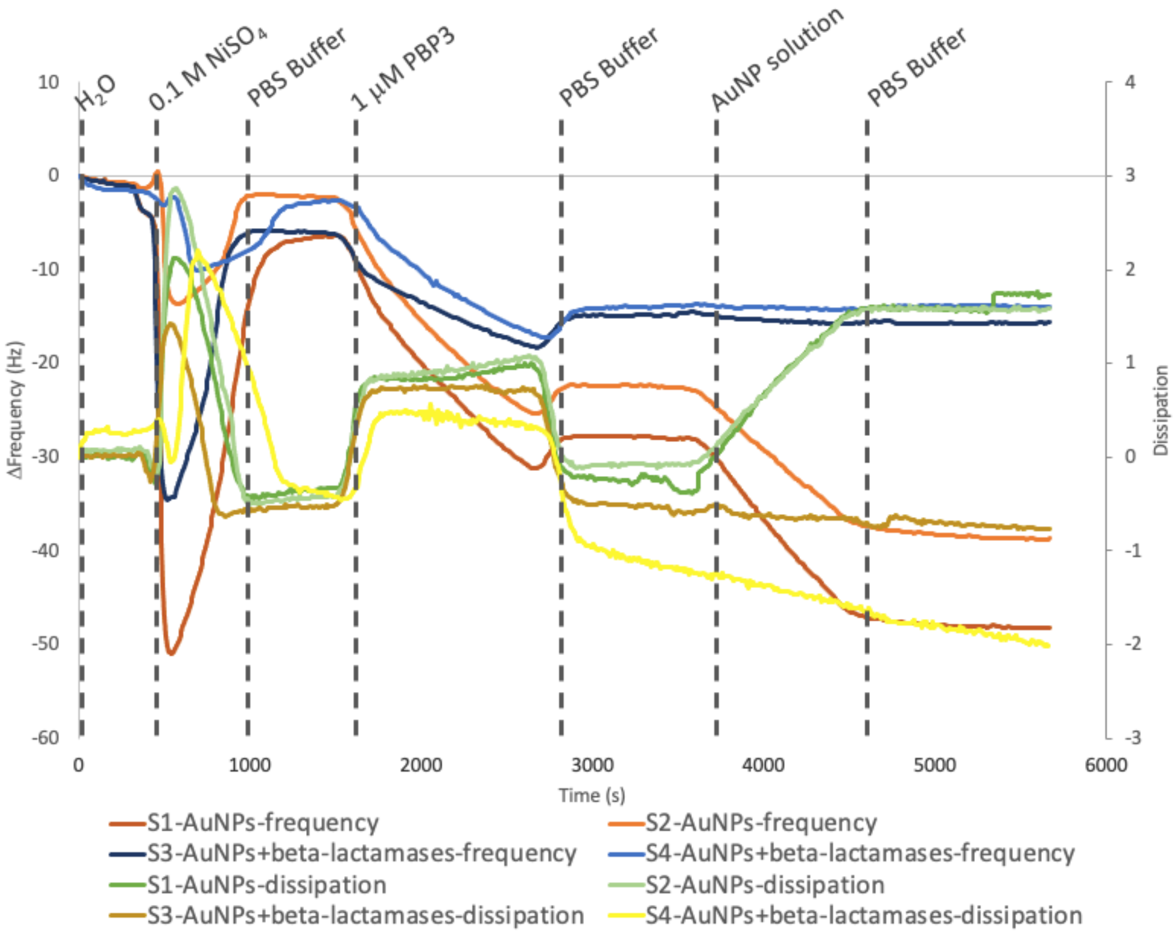

Fig. S6: QCM-D experiment with immobilization of PBP3 and introduction of antibiotic-functionalized AuNPs: No β-lactamase (S1 and S2) vs pre-exposed to β-lactamases (S3 and S4).

4. LFA Optimization

4.1. Membrane optimization

A total of 15 membranes were trialed using the antibiotic-functionalized AuNPs. All membranes were tested with three solutions of antibiotic-functionalized AuNPs: (i) 40 nm AuNPs in buffer; (ii) 20 nm AuNPs in buffer; (iii) 20 nm AuNPs in urine. For successful LFA running and binding of the antibiotic-functionalized AuNPs, the test was recorded as “Y”; if unsuccessful (streaking of AuNPs in membrane or issues binding), the test was recorded as “N”. Each membrane was then scored out of a possible 3, determined by the number of successful tests.

Five of the membranes performed well with the test samples (highlighted in green) and two membranes performed well with the 20 nm AuNPs (highlighted in orange). Further testing identified *HF090MC100* as the optimal membrane (data not shown).

Table S4. Test of nitrocellulose membranes

Membrane	40 nm AuNPs in buffer	20 nm AuNPs in buffer	20 nm AuNPs in urine	Score (3)
HF090MC100[a]	Y	Y	Y	3
Immunopore RP[b]	Y	Y	Y	3
Immunopore FP[b]	Y	Y	Y	3
Immunopore SP[b]	Y	Y	Y	3
Immunopore XP[b]	N	N	N	0
FF120HP[b]	N	Y	Y	2
FF120HP plus thick[b]	N	N	N	0
FF170HP plus thick[b]	Y	N	Y	2
FF80HP plus thick[b]	N	N	N	0
FF80HP[b]	N	N	N	0
FF170HP[b]	N	Y	Y	2
PRIMA40[b]	N	N	Y	1
FF170HP plus[b]	Y	Y	Y	3
FF80FP plus[b]	N	N	N	0
FF120HP plus[b]	N	N	N	0

Suppliers: [a]Merck; [b]Cytiva

4.2. PBP3 spotting in test zone of LFA

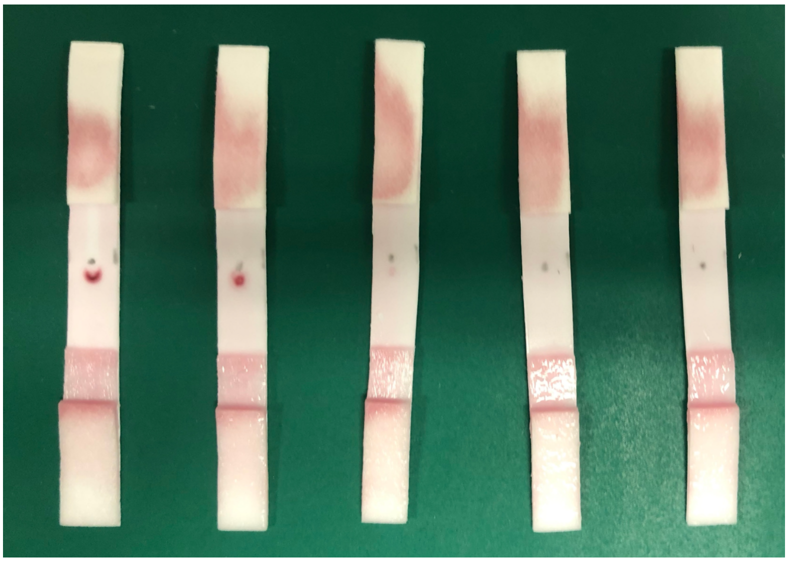

Fig. S7: From left to right, PBP3 concentration 10 μM, 1 μM, 100 nM, 10 nM, and 1 nM

4.3. Biotinylated-BSA spotting in control compatibility studies

Materials: Biotin, NHS, *N*,*N*'-dicyclohexylcarbodiimide (DCC), and BSA were purchased from Sigma Aldrich. All solvents were purchased from Fisher. All reagents and solvents were used as supplied.

Preparation of biotin-NHS: Biotin (100 mg, 0.410 mmol, 1 equiv.) was dissolved in DMF (3 mL, 0.1 M) by heating at 50 °C for 10 min. To this solution, NHS (47 mg, 0.410 mmol, 1 equiv.) was added and the reaction mixture was allowed to return to room temperature. Thereafter, DCC (110 mg, 0.534 mmol, 1.3 equiv.) was added and the reaction was stirred for a further 18 h at room temperature. The resultant suspension was filtered and the filtrate concentrated to a white solid (199 mg). Purification by column chromatography, eluting with 4-8% MeOH/DCM, afforded the biotin-NHS product as a while solid (95 mg, 68% yield).

^{1}H NMR (400 MHz, DMSO-d_6) δ 6.43 (s, 1H), 6.37 (s, 1H), 4.39 – 4.22 (m, 1H), 4.19 – 4.07 (m, 1H), 3.10 (q, J = 7.5, 6.8 Hz, 1H), 2.86 – 2.82 (m, 1H), 2.81 (s, 4H), 2.67 (t, J = 7.4 Hz, 2H), 2.58 (d, J = 12.3 Hz, 1H), 1.64 (p, J = 7.4 Hz, 3H), 1.56 – 1.37 (m, 3H). **HRMS**: exact mass calculated for [M+Na$^+$] ($C_{14}H_{19}N_3NaO_5S$) required *m/z* 364.0938, measured *m/z* 364.0934.

^{1}H consistent with reported data: K. Susumu *et al.*, *JACS*, **2007**, 129 (45), 13987 – 13996.

Preparation of biotinylated-BSA: Biotin-NHS (5 mg in 50 μL DMSO) was added to a solution of BSA in PBS (10 mg in 950 μL) then the mixture was shaken at room temperature for 1 h. Dilutions were prepared in PBS buffer as required.

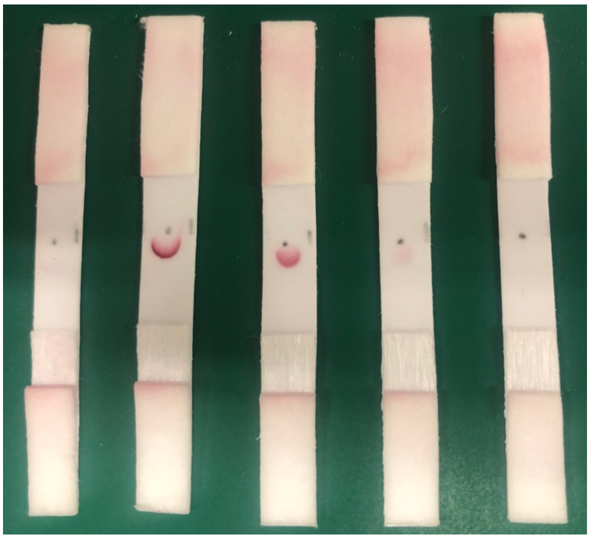

Fig. S8: From left to right, biotinylated-BSA concentration: 10 mg/mL, 1 mg/mL, 100 μg/mL, 10 μg/mL, and 1 μg/mL.

Note: 10 mg/mL solution: precipitate formation over time due to low solubility of biotinylated-BSA in PBS, supernatant used in pipetting of spot, but no binding of streptavidin-AuNPs observed likely due to insufficient biotinylated-BSA in the membrane.

5. LFA testing

5.1. Incubation Temperature Studies

Table S5. Effects of incubation temperature using CTX-M-15: Y = successful detection; N = no detection

CTX-M-15 concentration	20 °C	37 °C	50 °C
6 µM	Y	Y	NT[a]
600 nM	N	Y	Y
60 nM	N	Y	Y
6 nM	N	N	N
600 pM	N	N	N

[a] Not tested (NT) aggregation of AuNPs observed at higher temperature with higher concentration of CTX-M-15

5.2. Limit of detection *in vitro*

5.2.1. *In vitro* results with LFA

Concentrations were screen in triplicate, exemplar results are provided below. Enzymes were diluted in PBS or urine as required.
Note: The coloured test spots were easier to observe by eye than shown in the photos.

Table S6(a). Results of *in vitro* screen in buffer

β-lactamase	LFA Results in Buffer				
TEM-1	-	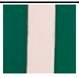 6 µM	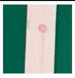 600 nM	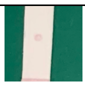 60 nM	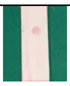 6 nM
AmpC	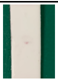 12 µM	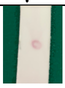 1.2 µM	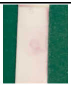 120 nM	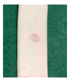 12 nM	-
CTX-M-1	-	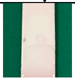 7.8 µM	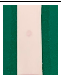 780 nM	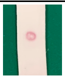 78 nM	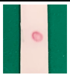 7.8 nM
CTX-M-15	-	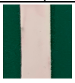 6 µM	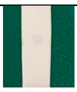 600 nM	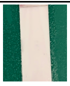 60 nM	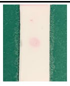 6 nM
NDM-1	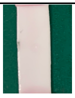 11 µM	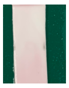 1.1 µM	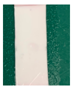 110 nM	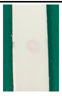 11 nM	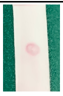 1.1 nM

Table S6(b). Results of *in vitro* screen in urine

β-lactamase	LFA Results in Urine				
TEM-1	-	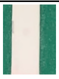 6 µM	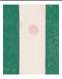 600 nM	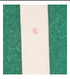 60 nM	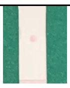 6 nM
AmpC	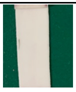 12 µM	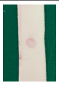 1.2 µM	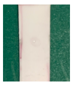 120 nM	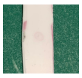 12 nM	-

**CTX-M-1**	-	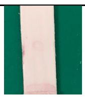 7.8 μM	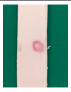 780 nM	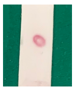 78 nM	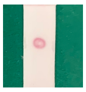 7.8 nM
**CTX-M-15**	-	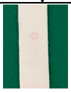 6 μM	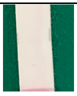 600 nM	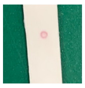 60 nM	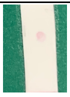 6 nM
**NDM-1**	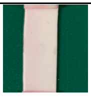 11 μM	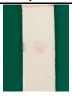 1.1 μM	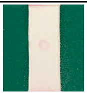 110 nM	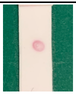 11 nM	-

5.2.2. *In vitro* results with CTX-M and CTX-M M

CTX-M and CTX-M M LFAs were purchased from Una Health Ltd, UK and used according to the suppliers protocol. Concentration screen was carried out using recombinant CTX-M-15. In each test, the top line is the control and the lower line is the test.

Results found that both the CTX-M and CTX-M M LFAs were able to detect CTX-M-15 at the lowest concentration tested: 1.2 fM.
Note: The coloured test lines were easier to observe by eye than shown in the photos.

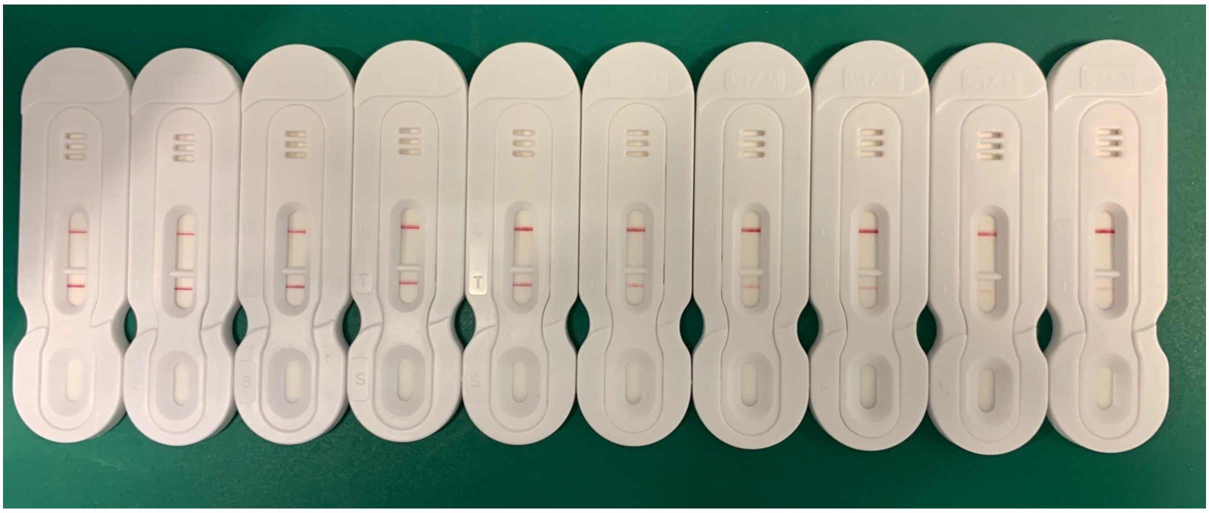

Fig S9: Results of CTX-M *in vitro* screen: Concentration range of 1.2 μM (left) – 1.2 fM (right), incremental serial dilutions of 10-fold.

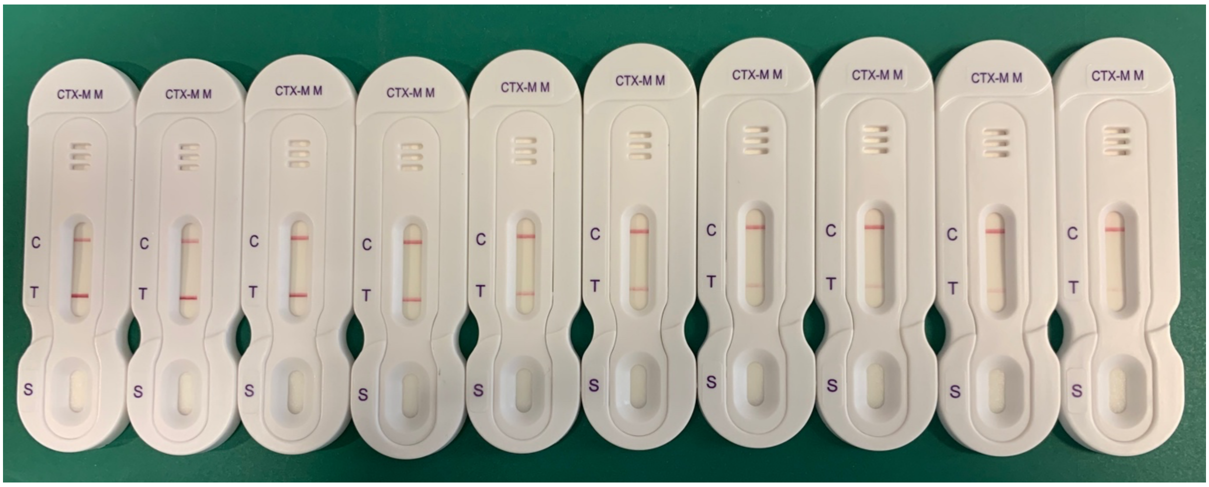

Fig S10: Results of CTX-M M *in vitro* screen: Concentration range of 1.2 μM (left) – 1.2 fM (right), incremental serial dilutions of 10-fold.

6. Compatibility with Hematuria

Samples were prepared by spiking urine with human blood (BCR®, certified research material, Sigma Aldrich, BCR635).

Table S7. Effects of blood present in urine sample.

Blood content in urine (%v/v)	Observation
5	Poor reproducibility, some aggregation of AuNPs
2.5	Good binding in LFA
1.25	Good binding in LFA

7. Correlation of β-lactamase concentration

7.1. Nitrocefin assay protocol

Bacterial samples (1 mL) were centrifuged at 10,000 x g for 10 minutes and 950 μL of the supernatant was discarded. The pellet was then washed by re-suspending with the addition of 950 μL PBS, centrifuging and discarding the supernatant (950 μL). The pellet was then lysed by the addition of 950 μL BugBuster® and allowing to incubate at room temperature for 15 min. The samples were then centrifuged at 16,000 x g at 4 °C for 20 minutes to remove any debris and the supernatant transferred to a fresh tube. Aliquots of 50 μL from the samples were added into wells in triplicate. Next 50 μL of the nitrocefin test solution (prepared according to the Sigma Aldrich commercial kit protocol) was then added, gently mixed by pipette, and the hydrolysis monitored by measuring the absorbance at 490 nm. Controls of BugBuster® confirmed no interference with the assay. Blanks of nitrocefin in the absence of β-lactamases were included to measure the background hydrolysis.

The absorbance (490 nm) for each well was plotted versus time, corrected for the background by subtracting the measurement obtained for the blank nitrocefin controls. Using calibration curves obtained using standards of hydrolyzed nitrocefin, the reaction rate between two set time points was determined and the β-Lactamase

activity in sample well calculated in nmole/min/mL (milliunits/mL). The β-lactamase concentrations of the lysates were then determined using standard curves of β-lactamase activity determined using pure recombinant β-lactamases of known concentration. Each triplicate is plotted in Fig. S8 and S9 vs. the CFU/mL determined for the bacterial sample.

7.2. CFU/mL Determination

The bacterial samples used in the nitrocefin assays were grown on LB agar plates to determine the CFU/mL for each sample. Three aliquots were taken from each sample and plated, the average CFU/mL for these three plates was used as the CFU/mL for the associated bacterial sample.

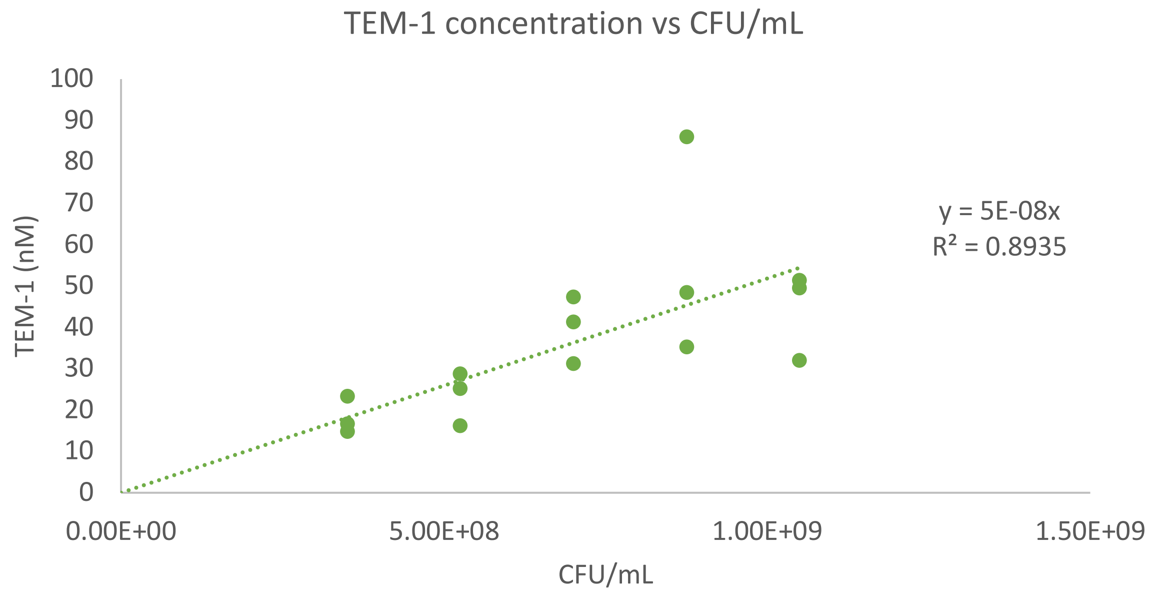

Fig. S11: Measured TEM-1 concentrations vs. CFU/mL

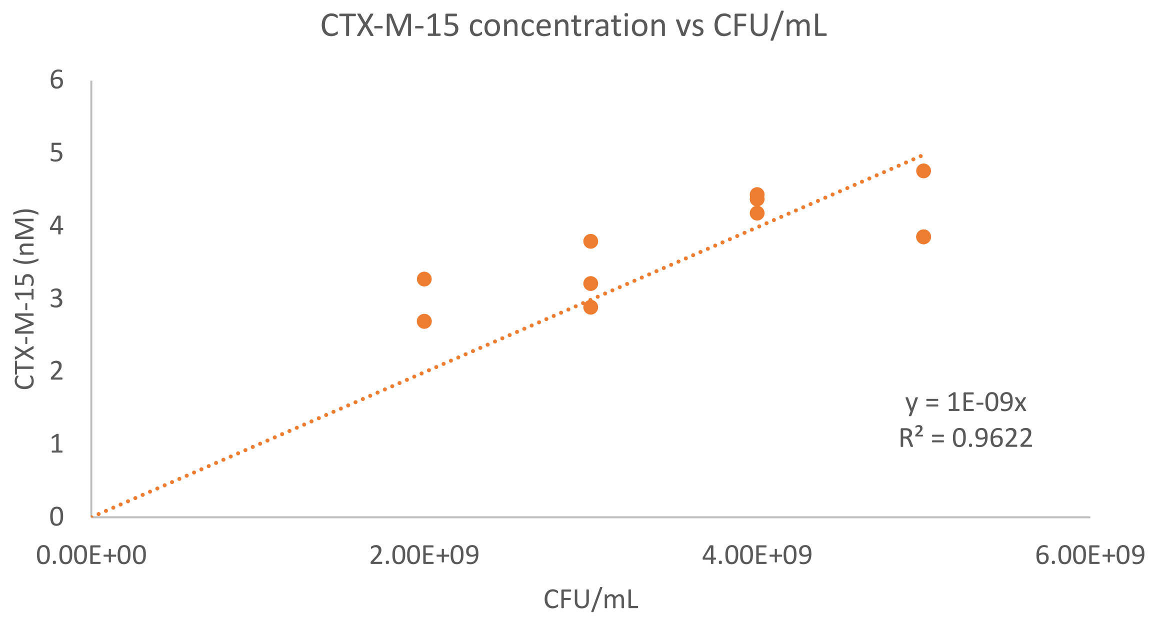

Fig. S12: Measured CTX-M-15 concentrations vs. CFU/mL

Table S8. β-lactamase concentration ranges in typical UTI samples.

**Bacterial strain, β-lactamase**	**β-lactamase concentration in lysate of UTI sample of 10^4-10^6 CFU/mL**
E. coli (ATCC 35218), TEM-1	250 fM - 25 pM
E. coli (BW25113), CTX-M-15	600 fM - 60 pM
